# Comparison of Dexamethasone 4mg vs 8mg Doses in Total Joint Arthroplasty Patients: A Retrospective Analysis

**DOI:** 10.7759/cureus.10295

**Published:** 2020-09-07

**Authors:** Sivasenthil Arumugam, Katherine Woolley, Ryan A Smith, Smitha Vellanky, Michael S Cremins, Latha Dulipsingh

**Affiliations:** 1 Anesthesiology, St. Francis Hospital and Medical Center, Hartford, USA; 2 Anesthesiology, University of Connecticut School of Medicine, Farmington, USA; 3 Orthopedics, Frank H. Netter MD School of Medicine, Quinnipiac University, North Haven, USA; 4 Connecticut Joint Replacement Institute, St. Francis Hospital & Medical Center, Trinity Health of New England, Hartford, USA; 5 Diabetes and Endocrinology, St. Francis Hospital & Medical Center, Trinity Health of New England, Hartford, USA

**Keywords:** diabetes, ponv, postop pain, perioperative glucose, glucocorticoids, perioperative medicine, patient outcomes, multimodal analgesia, dexamethasone doses

## Abstract

Introduction

Dexamethasone is commonly administered intraoperatively to control postoperative nausea and vomiting (PONV) and pain. There is limited evidence of the ideal dosage of dexamethasone during surgery. Dexamethasone administration may increase blood glucose levels, posing unique challenges in maintaining acceptable blood glucose levels in patients with diabetes.

Objective

Compare two doses of dexamethasone (4mg and 8mg) for outcomes in patients undergoing hip and knee arthroplasty.

Methods

Medical records of 3,194 patients having undergone total hip arthroplasty (THA) and total knee arthroplasty (TKA) between January 1, 2016 and December 31, 2017 who were administered dexamethasone were reviewed. The eligible population included male and female patients aged 18-89, who underwent elective hip and knee replacement surgery and were administered dexamethasone intraoperatively. Demographics, clinical variables, and preoperative diabetic status were recorded. Primary outcomes included: blood glucose levels, incidence of PONV, post-anesthesia care unit (PACU) time, and length of stay (LOS). Postoperative complications such as periprosthetic joint injection and urinary tract infections (UTI) were also examined within 90 days of surgery. The 30-day readmissions rate was also collected for analysis.

Results

There was no PONV in the entire patient population. There were no significant differences between 4mg and 8mg dexamethasone in patients with or without diabetes, for preop to postop blood glucose difference, surgical timing, and post-operative complications.

Conclusion

Dexamethasone in both 4mg and 8mg dose was effective in PONV prophylaxis. The effects of 4mg and 8mg dexamethasone were the same in individuals with and without diabetes and the increases in blood glucose were not significantly different. Dexamethasone should not be withheld, as its benefits seem to outweigh the risks even in patients with diabetes.

## Introduction

General anesthesia and intravenous sedation for surgical procedures are associated with a 20-30% incidence of postoperative nausea and vomiting (PONV) [1-2]. Improvements in surgical and anesthetic management have made total joint arthroplasties (TJA) more efficient and tolerable for patients. Recently effective control of pain and PONV with innovative care pathways has resulted in improved outcomes like quicker discharge and better patient satisfaction.

Dexamethasone is a synthetic corticosteroid with high potency and a long duration of action (half-life of two days), used in TJA and other surgical procedures for its analgesic and antiemetic effects [1-8]. Perioperative intravenous (IV) administration of dexamethasone has been shown to decrease pain scores, reduce postoperative opioid consumption, and increase patient satisfaction, in addition to reduction in PONV. Dexamethasone use has been shown to shorten hospital stays, expedite recovery, and reduce healthcare costs [1-4,6,8-12]. There are multiple sites of action at which glucocorticoid-activated receptors produce anti-inflammatory and immunosuppressive effects. It is not very clear as to the mechanism of action of the glucocorticoids in reducing pain and PONV. Prostaglandins are one of the main inducers of inflammation after tissue injury, and glucocorticoids reduce prostaglandin synthesis by inhibiting the expression of Cox-2. Several studies also note a reduction in postoperative inflammatory markers and C-reactive protein levels in patients who received dexamethasone [6,13-15]. For these reasons, it is now an integral part of multimodal regimen for both, PONV prophylaxis and perioperative analgesia.

Administration of dexamethasone raises blood glucose levels posing unique challenges in patients with diabetes. For diabetic patients, there is conflicting evidence regarding whether or not there is a dose dependent response in the elevation of blood glucose levels [16-18]. The anti-inflammatory nature of corticosteroids, through inhibition of prostaglandin synthesis, may also have detrimental side effects with high or repeated dosing. Undesirable side effects may include hyperglycemia, altered immune responses and delayed wound healing. There is lack of consensus and limited evidence available for an effective dose of dexamethasone without significant adverse effects. Commonly administered dose of perioperative dexamethasone ranges anywhere from 4 mg to 25 mg [1-3]. At our institute, most practitioners administer either 4mg or 8mg dose. As there is no conclusive evidence for optimal dexamethasone dose especially in diabetic patients, we analyzed the data comparing the doses for benefits and side effects to further guide our practice. The purpose of this retrospective study was to compare two doses of dexamethasone (4mg and 8 mg) in patients undergoing total joint arthroplasty (TJA).

## Materials and methods

This is a retrospective study to measure clinical and outcome metrics in patients admitted to Saint Francis Hospital and Medical Center who underwent elective TJA between January 1, 2016 and December 31, 2017 and received dexamethasone intraoperatively. After obtaining approval from Institutional Review Board (IRB # SFH-18-116), medical records of 3,194 patients having undergone hip or knee arthroplasty between January 1, 2016 and December 31, 2017 who were administered dexamethasone were obtained from the Connecticut Joint Replacement Institute (CJRI) registry. The eligible population was male and female patients aged 18 to 89 years, who underwent elective hip and knee surgery and were administered a single dose of dexamethasone intraoperatively. We excluded patients who received dexamethasone more than once on the day of surgery, who received dexamethasone doses other than 4 mg or 8 mg and who did not have blood glucose measured preoperatively and postoperatively on the day of surgery. This resulted in eliminating more than 2400 patients, but limited the confounding variables, data and the groups comparable. There were a total of 715 patients who met the criteria and were included in this analysis. Diabetic status was determined through a diagnosis of diabetes in history and was recorded as either diabetic (D) or non-diabetic (ND). Data regarding demographics (Table 1), clinical variables, and preoperative diabetic status were recorded. Primary outcomes included: blood glucose levels, incidence of postoperative nausea and vomiting (PONV), post-anesthesia care unit (PACU) time, and length of stay (LOS). Postoperative complications such as periprosthetic joint infection (PJI) and urinary tract infections (UTI) were also examined within 90 days of surgery. PJI diagnosis was made as per Musculoskeletal Infection Society (MSIS) criteria. A post hoc power calculation determined adequate power. Continuous variables such as postop blood glucose levels and length of stay were assessed for normality and differences between groups using Kruskal-Wallis test. Categorical variables were tested using the Pearson Chi-Square test. All analysis was conducted using Statistical Package for the Social Sciences (SPSS) software, version 25 (IBM Corp., Armonk, NY).

## Results

A total of 715 patients were analyzed, of which 20.7% received 4mg dexamethasone and 79.3% received 8mg dexamethasone. Majority of the patients (78.4%) who received 4mg dexamethasone were patients with diabetes, while the percentage of those with diabetes was slightly lower (61.6%) among patients receiving 8mg dexamethasone. Patients receiving 4mg were slightly older than those receiving 8mg (4mg: 70.4 + 8.92, 8mg: 67.8 + 9.00; p = 0.003). On average, there were no differences in sex distribution or BMI between patients receiving 4mg versus 8mg dexamethasone (Table 1).

**Table 1:** Subject Demographics * indicates a statistically significant difference between the two groups.

	4mg	8mg
N	148 (20.7%)	567 (79.3%)
Patients with Diabetes	116 (78.4%)	349 (61.6%)
Patients without Diabetes	32 (21.6%)	218 (38.4%)
Age (yrs ± SD)	70.4 + 8.92*	67.8 + 9.00
Sex (M/F)	52.7%/47.3%	48.5%/51.5%
BMI (Mean ± SD)	33.6 + 6.16	32.5 + 5.95

Surgical timing (active operative time and total time in the OR), PACU time, and length of stay were not significantly different between the two groups (Table 2). Both preoperatively and postoperatively the blood glucose levels were significantly higher in the 4mg group (Table 3). However, no differences were observed in preoperative to postoperative blood glucose change between groups (Table 3).

**Table 2:** Comparison of time for both groups OR: Operating room; PACU: Post-anesthesia care unit; LOS: Length of stay; Hrs: Hours; SD: Standard deviation.

	4mg	8mg
OR Time Hrs (Mean ± SD)	2.2 + 0.70	2.3 + 0.71
PACU Time Hrs (Mean ± SD)	2.5 + 0.89	2.5 + 1.07
LOS days (Mean ± SD)	2.3 + 1.01	2.3 + 1.33

**Table 3:** Blood glucose values for both groups * Indicates a statistically significant difference between the two groups.

	4mg	8mg
Preop Blood Glucose (Mean ± SD) mg/dL	134.89 + 32.216*	122.95 + 28.300
Postop Blood Glucose (Mean ± SD) mg/dL	161.96 + 43.998*	152.33 + 40.156
Change in Preop to Postop blood glucose (Mean ± SD)	27.07 + 42.577	29.38 + 32.121

There were no incidences of PONV observed in the overall study population. Additionally, no differences between groups were observed in UTI rate, PJI rate or rates of 90-day complications or 30-day readmissions.

Among the diabetic patient population, patients receiving 4mg versus 8mg dexamethasone were similar in demographics and surgical times. Diabetic patients who received 8mg dexamethasone had significantly lower preoperative blood glucose than those that received 4mg. However, postoperative blood glucose levels and change in preoperative to postoperative blood glucose levels were comparable between the groups.

Insulin bolus dose (regular Insulin sliding scale as per CJRI guidelines) was administered in five patients intraoperatively and 38 patients postoperatively in PACU. All who received insulin were patients with diabetes. Among five patients who needed insulin intraoperatively, only one patient received 4 mg dexamethasone and four patients received 8 mg dexamethasone. In the PACU among 38 patients who needed insulin, 10 patients were from 4 mg group and 28 patients were from 8 mg group. Only one patient received insulin both intraoperatively and the PACU and was from the 8 mg group. 7.43% of patients with diabetes from 4 mg group required insulin and 5.46% of patients with diabetes from 8 mg group required insulin.

None of the patients had any incidence of hypoglycemia intraoperatively or in the PACU. No differences were observed in any metrics among non-diabetic patients who received 4mg versus 8mg dexamethasone.

## Discussion

Intraoperative administration of dexamethasone has been shown to improve outcomes such as PONV and pain [1-7]. The aim of this study was to determine the difference between 4mg and 8mg doses of perioperative dexamethasone with regards to PONV, blood glucose, surgical time and complications.

None of the patients in our analysis had any incidence of PONV, suggesting that there may be no difference between 4mg and 8mg dexamethasone for PONV prophylaxis. Recent consensus guidelines and a meta-analysis of randomized clinical trials also recommend 4mg dose of dexamethasone for effective PONV prophylaxis [19,20]. In our study population about 65% patients who received 4mg and about 60% patients who received 8mg dexamethasone had spinal anesthesia as primary anesthetic. Even though PONV is more common among those who receive general anesthetic, we did not analyze these subgroups as none of the patients had PONV.

As this is a retrospective study, data collected was not adequate to analyze the effect on post-operative pain. There is an abundance of literature now supporting the analgesic effects of dexamethasone. A meta-analysis of dexamethasone for pain management for patients undergoing total knee arthroplasty found that dexamethasone treatment reduced postoperative pain scores, opioid use at 12 hours post-op, and decreased complication rate. The analysis included intravenous doses ranging from 10mg to 20mg [21]. Additional studies have found decreased utilization of antiemetic and analgesic medications postoperatively and decreased LOS in patients administered 8mg to 10mg intravenous dexamethasone without increasing complications [10,22]. Kardash et al. found that a single, preoperative dose of 40mg dexamethasone led to decreased PONV and dynamic pain following total hip arthroplasty [23]. Additionally dexamethasone appears to demonstrate a dose effect. Oliveira et al. compared the effects of 0.05 mg/kg and 0.1 mg/kg dexamethasone administered before induction in outpatient gynecological surgery. They found that the larger dose provided greater pain relief and improved recovery [24]. Marhofer et al. found no clinical analgesic benefit to adding dexamethasone (either intravenous or perineural) and one of the reasons could be that a dose of 4mg may be inadequate for prolonging pain relief [25].

Although there are many benefits to perioperative administration of dexamethasone, there are concerns about increased risk of undesirable outcomes like hyperglycemia and infections. Intraoperative hyperglycemia is a known phenomenon and intraoperative administration of dexamethasone may contribute or exaggerate this. Contrary to the belief that a larger dose of dexamethasone would lead to greater increases in post-op blood glucose, our study showed no significant difference between the two doses. The increase in preoperative to postoperative blood glucose between 4mg or 8mg of dexamethasone (27.07 ± 42.57 vs 29.38 ± 32.12) among our patients is shown in Table 3. The proportion of patients requiring insulin was not higher in 8 mg group (5.46%) compared with 4 mg group (7.43%) in our study.

Additionally, we anticipated patients with diabetes to have an exaggerated response to administration of dexamethasone, but there was again no significant difference in preoperative to postoperative blood glucose when comparing patients with diabetes and without diabetes. This indicates that both 4mg and 8mg intraoperative dexamethasone have a similar effect on postoperative blood glucose. This is contrary to a study by Abdelmalak et al. who found that dexamethasone caused significantly increased postoperative blood glucose in patients without diabetes, but not in patients with diabetes. One possible cause for this is that this study treated blood glucose >215 mg/dL with insulin, potentially decreasing the change from preoperative to postoperative blood glucose [26]. They postulate that smaller doses of dexamethasone (4-8mg) such as in our study are less likely to significantly increase blood glucose and should still be considered for controlling PONV and pain [26]. In our study patients with diabetes who required insulin were not higher in 8 mg group (5.46%) compared to 4 mg group (7.43%). Several other studies have demonstrated similar results to ours. Nurok et al. conducted a retrospective study investigating perioperative dexamethasone administration in patients undergoing TJA and found that there was no increased risk of having a post-op blood glucose >200 mg/dl in patients given dexamethasone when compared to patients who were not [27]. They also reported no difference in infection and wound healing.

Numerous studies assessing wound healing and infection rates found no difference in results between IV dexamethasone groups and non-dexamethasone recipients even when patients with diabetes were included in these groups [2-4,8,15,28]. There was also no difference noted for infections related specifically to TJA patients [1,29,30].

This supports our study, as we found no significant increases in blood glucose between the 4mg and 8mg of dexamethasone, regardless of diabetic status. This may allow for less intensive post-operative blood glucose monitoring and insulin requirements, facilitating arthroplasties to be performed as an outpatient procedure. Additionally, there were no differences in surgical timing and complication rates. As this is a retrospective study with its inherent limitations, the findings need to be confirmed with a prospective randomized trial.

## Conclusions

Intraoperative dexamethasone in both 4mg and 8mg dose adequately controlled PONV. Given that we found no difference in preoperative to postoperative blood glucose, surgical timing, or adverse events in the 4mg versus 8mg dexamethasone groups, both appear to be safe options, with 8mg possibly offering greater pain relief. There was no difference in these parameters between patients with or without diabetes. Therefore, perioperative dexamethasone should not be withheld from patients with diabetes for fear of hyperglycemia or risk of infection as the benefits of PONV prophylaxis and pain control appears to outweigh the risks.
